# Exploring the Symptoms and Treatment of a Pituitary Macroadenoma: A Case Report

**DOI:** 10.7759/cureus.85640

**Published:** 2025-06-09

**Authors:** Nishat Jahan Chowdhury, Miral S Abu Rumman, Md Shadman Tawsif Zaman, Shiju Shikder, Tawhida Jahan Youshra, Maria Khan, Sadia Bano

**Affiliations:** 1 Medicine, Sylhet Women's Medical College and Hospital, Sylhet, BGD; 2 Faculty of Medicine, University of Jordan, Amman, JOR; 3 Intensive Care Unit, Evercare Hospital, Dhaka, BGD; 4 General Medicine, NHS (National Health Service) Forth Valley, Larbert, GBR; 5 General Medicine, King’s College Hospital, London, GBR; 6 Emergency Medicine, Asian Institute of Gastroenterology, Hyderabad, IND; 7 Medicine, Nishtar Medical University, Multan, PAK

**Keywords:** case report, imaging modulities, pituitary macroadenoma, symptoms, treatment

## Abstract

Pituitary macroadenoma (PMA) are significant intracranial tumors that can cause local mass effects and systemic endocrine disruptions. This case report illustrates an unusual case of PMA in a patient presenting with an altered level of consciousness subsequently coupled with headache and throat pain. A 78-year-old man reported to the emergency room with a history of an altered level of consciousness followed by headache and throat pain for two days. He had a past medical history of diabetes mellitus (DM), hypertension (HTN), severe hypothyroidism, ischemic heart disease, cholelithiasis, and right vesicoureteric junction calculus. The physical examination yielded minimal findings, with no indications of hypogonadism, acromegaly, or hyperprolactinemia. Magnetic resonance imaging (MRI) identified a PMA measuring 1.5 × 2.2 × 2.3 cm extending from the sella turcica with suprasellar expansion next to the optic chiasm and into the sphenoid sinus. Laboratory tests showed decreased sodium and adrenocortical hormone (ACTH) levels. Due to the size of the tumor and the patient's condition, surgical intervention was not undertaken. The patient was managed with continued pharmacological treatment, including vaptan, follow-up imaging, and endocrinological monitoring. This case emphasizes the importance of considering macroadenomas in differential diagnosis when encountering unusual patient presentations with a complex medical history. Therefore, early diagnosis and individualized management are crucial for achieving optimal outcomes.

## Introduction

Pituitary adenomas are benign intracranial tumors that arise from the anterior pituitary lobe and account for 15% of the primary intracranial tumors. They are either classified (according to their size) as micro- or macroadenomas or (according to hormone secretion) as secretory or non-secretary adenomas [1,2]. Pituitary adenomas are considered macroadenomas when the neoplastic mass has a diameter that is more than 10 mm. They are clinically significant due to their propensity to compress adjacent structures in the sellar and parasellar areas. In addition, these tumors may sometimes become invasive and involve structures beyond the sellar area, such as the internal carotid, nasal cavity, sphenoid, and cavernous sinuses [3]. These tumors are considerably rare. Their incidence is estimated to be 25 cases per 100,000 individuals [2]. The clinical presentation of these tumors usually varies. Patients may present with symptoms of a local mass effect such as headache, visual disturbances, and cranial nerve palsies, while others present with symptoms of endocrine dysfunction such as unexplained hair growth or loss or erectile dysfunction. The diagnostic approach of pituitary adenomas usually varies based on the symptoms that the patient presents with. Various radiological modalities, such as magnetic resonance imaging (MRI) and computed tomography scans (CT) are ordered to assess the tumor's location, its size, and how it affects adjacent structures. Meanwhile, laboratory studies are requested to assess hormone levels to detect the presence of any endocrinal dysfunctions. Surgical intervention is usually the primary intervention to treat pituitary macroadenoma (PMA) tumors as surgeons gain access to the pituitary gland using the transsphenoidal approach [4,5]. Herein, we report a case of a 78-year-old man who presented to the emergency room with atypical symptoms of PMA, which was a loss of consciousness and followed a different treatment plan, not surgical intervention, that suited the patient’s health.

## Case presentation

A 78-year-old man presented to the emergency room because he had been experiencing a two-day altered level of consciousness, followed by a headache and throat pain for the same duration. His past medical history included diabetes mellitus (DM), hypertension (HTN), ischemic heart disease, cholelithiasis, and right vesicoureteric junction (VUJ) calculus. The patient denied acute or chronic stress in his life or a past history of similar conditions.

**Figure 1:**
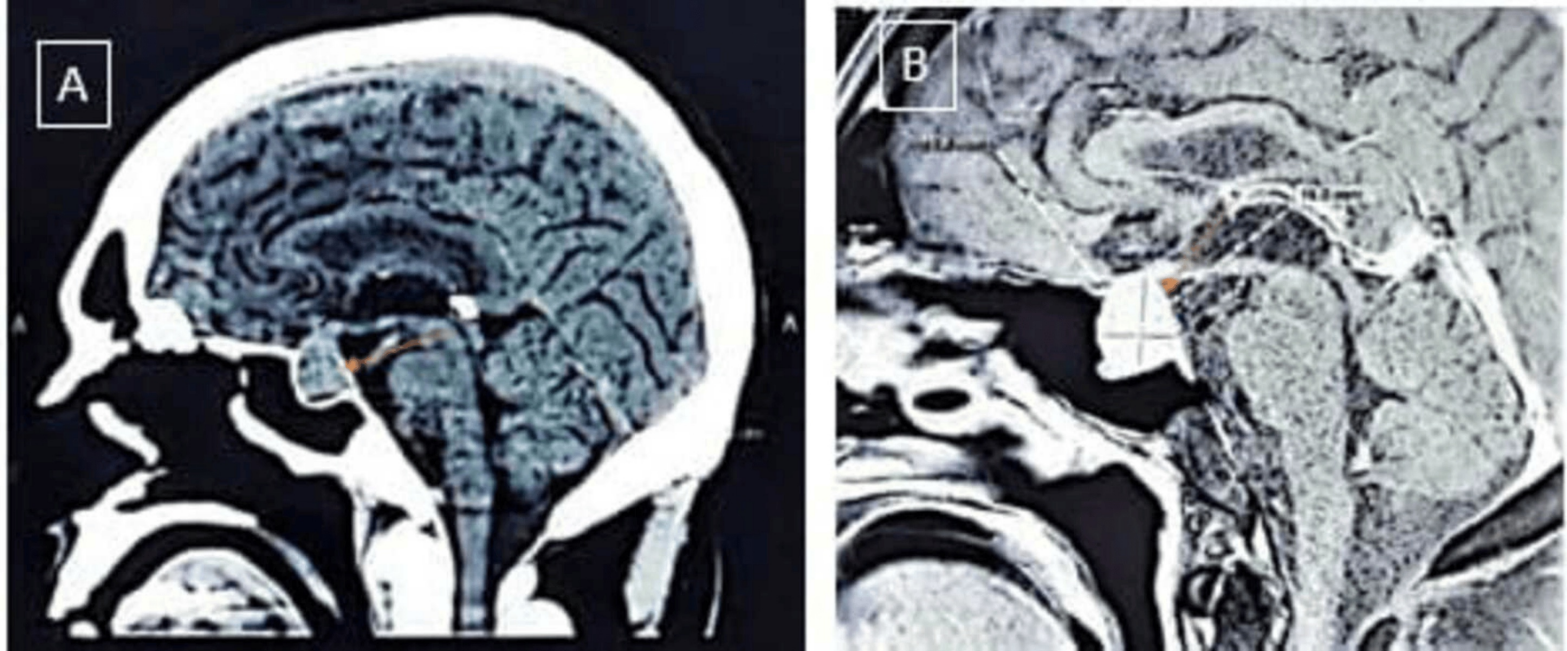
Sagittal view of imaging studies depicting a sellar mass. Panel A displays a CT image, while panel B shows an MRI image. Both images highlight the mass’s extension into the suprasellar region and sphenoid sinus.

Upon physical examination, the patient looked reasonably well and was alert and oriented to time, place, and person. His blood pressure (BP), heart rate, temperature, and oxygen saturation (SpO2) were 140/80 mm of Hg, 78 beats/min, 98 F, and 98% in room air, respectively. His random blood sugar (RBS) was 14.6 mmol/l. His respiratory, cardiovascular, and abdominal exams were unremarkable; he had a vesicular breath sound with no added sounds. S1 and S2 were heard with no added sounds and a soft, nontender abdomen with no palpable organomegaly. The bowel sound was present. The clinical neurological examination was unremarkable; he scored E4V5M6 on the Glasgow Coma Scale (GCS) with intact cranial nerve and sensory function. His muscle power was as follows: right upper limb (RUL) 4/5, right lower limb (RLL) 3/5, left upper limb (LUL) 4/5, and left lower limb (LLL) 3/5. There was no clinical evidence of acromegaly or hypogonadism. An MRI and a CT scan of the brain and laboratory blood tests were conducted to evaluate the condition extent further. The imaging confirmed the presence of a lobulated mass (1.5 x 2.2 x 2.3 cm in size). The tumor originated in the sella turcica region with suprasellar extension abutting the optic chiasm and inferior extension into the sphenoid sinus (Figure 1).

Blood tests showed a slightly decreased adrenocorticotropic hormone (ACTH) level of 1.77 pg/mL, serum cortisol level of 8.22 μg/dL, growth hormone 0.17 ng/mL, thyroid stimulating hormone (TSH) level of 2.81 μlU/mL, serum prolactin level 159 mlU/L, serum follicle-stimulating hormone (FSH) level 2.96 mlU/mL, serum luteinizing hormone (LH) level 0.829 mlU/mL, sodium level of 129 mmol/L, chloride level of 91 mmol/L, potassium level of 3.6 mmol/L (Table 1). The patient was started on Vaptan 15 mg within 24 hours of presentation to the hospital.

**Table 1:** Results of laboratory tests along with the corresponding reference values for each parameter. The reference values are the typical ranges observed in healthy individuals and can vary slightly depending on factors such as age, sex, and laboratory methods. ACTH: adrenocorticotropic hormone; GH: growth hormone; TSH: thyroid-stimulating hormone; PRL: prolactin, FSH: follicle-stimulating hormone; LH: luteinizing hormone; Na⁺: sodium, Cl⁻: chloride.

Test	Result	Normal value	
ACTH	1.77 pg/mL	5.0-46.0 pg/mL	
Serum cortisol	8.22 μg/dL	4.45-22.7 μg/dL	
GH	0.17 ng/mL	Less than 5 in men, less than 10 in women	
TSH	2.81 μlU/mL	0.30-4.5 μlU/mL	
PRL	159 mlU/L	78-380 mlU/L in men, 64-395 mlU/L in women	
FSH	2.96 mlU/mL	0.95-11.95 mlU/mL in men	
LH	0.829 mlU/mL	1.14 - 8.75 mlU/mL	
Na^+^	129 mmol/L	134-145 mmol/L	
Cl^-^	91 mmol/L	97-107 mmol/L	
Potassium level	3.6 mmol/L	3.6-5.2 mmol/L	

After being diagnosed with pituitary macroadenoma, the patient was referred to the neurosurgery clinic. Their recommendation (considering tumor size, site, and patient condition) was not to proceed with the surgical intervention for his macroadenoma and to continue his usual pharmacological treatment with imaging and endocrinological follow-up.

## Discussion

This case report highlights an atypical presentation of a PMA in an elderly male, whose initial symptoms included altered consciousness, headache, and throat pain. Unlike the more common presentations of visual disturbances or hormonal imbalances seen in such tumors, this patient exhibited neurological symptoms likely associated with hyponatremia, evidenced by decreased sodium levels and marginally low cortisol and ACTH. This atypical presentation underscores the need for thorough differential diagnosis, especially in elderly patients with a complex medical history, where initial symptoms may not point directly to the underlying condition [1].

PMA can manifest through a variety of symptoms depending on their size, location, and functional status [6]. When located near the optic chiasm, these tumors often lead to visual impairments, while hormone-secreting tumors may present with symptoms related to hyperprolactinemia, acromegaly, or Cushing's syndrome [1,7]. However, the patient’s MRI findings showed a suprasellar extension without compressive effects on the optic pathways, which explains the absence of visual deficits. This highlights the unpredictable clinical course of macroadenomas, where even significant anatomical changes may not always correlate with expected symptoms [2].

The standard treatment for symptomatic PMA involves surgical resection, particularly using a transsphenoidal approach, which effectively reduces tumor mass and relieves symptoms associated with compression [1,8]. However, treatment decisions should be individualized, taking into account factors such as patient age, comorbid conditions, and overall risk assessment [4]. In this case, the decision to avoid surgery and opt for pharmacological management was influenced by the patient’s advanced age and preexisting health conditions, including DM, HTN, and ischemic heart disease. Additionally, the tumor's stable appearance on imaging justified a more conservative approach [2]. Current guidelines support the consideration of non-surgical management for patients who may not be ideal surgical candidates, emphasizing regular monitoring and endocrine assessments to track tumor behavior and patient health [4,9].

The use of Vaptan was aimed at correcting the patient's hyponatremia, reflecting a key aspect of the treatment, that is, addressing symptomatic electrolyte imbalances that could have contributed to his altered consciousness. This case illustrates the importance of a multidisciplinary approach, involving endocrinologists, neurosurgeons, and radiologists, to provide comprehensive care [1]. Collaboration among these specialties ensures that decisions are made with a holistic understanding of the patient’s condition, balancing immediate therapeutic needs with long-term outcomes [2].

This case reinforces the need for clinicians to maintain a high index of suspicion for PMA, even when encountering less typical presentations [1]. It also highlights that individualized treatment plans, especially in older patients with comorbidities, can often provide a more favorable risk-benefit profile compared to aggressive interventions. Regular follow-ups with imaging and laboratory assessments are crucial to ensure timely adjustments to the management plan if the tumor shows signs of progression or if new symptoms emerge [2].

## Conclusions

This case underscores the importance of individualized treatment plans, balancing the risks and benefits of surgery versus conservative management in elderly patients with multiple health issues. It emphasizes the need for a multidisciplinary approach involving endocrinologists, neurosurgeons, and radiologists to ensure optimal care. And clarify that regular follow-ups with imaging and endocrinological evaluations are essential to monitor for tumor progression or the development of new symptoms that may require a different treatment approach.
